# Three-Dimensional Culture of *Rhipicephalus* (*Boophilus*) *microplus* BmVIII-SCC Cells on Multiple Synthetic Scaffold Systems and in Rotating Bioreactors

**DOI:** 10.3390/insects12080747

**Published:** 2021-08-19

**Authors:** Michael T. Suderman, Kevin B. Temeyer, Kristie G. Schlechte, Adalberto A. Pérez de León

**Affiliations:** 1 Cell Systems-3D LLC, League City, TX 77573, USA; msuderman@cellsystems3D.com; 2 Knipling-Bushland U.S. Livestock Insects Research Laboratory and Veterinary Pest Genomics Center, United States Department of Agriculture, Agricultural Research Service, 2700 Fredericksburg Road, Kerrville, TX 78028, USA; kristie.schlechte@usda.gov; 3 San Joaquin Valley Agricultural Sciences Center, United States Department of Agriculture, Agricultural Research Service, 9611 S. Riverbend Avenue, Parlier, CA 93648, USA; beto.perezdeleon@usda.gov

**Keywords:** *Rhipicephalus* (*Boophilus*) *microplus*, three-dimensional (3-D) cell culture, synthetic scaffolds, ticks

## Abstract

**Simple Summary:**

Ticks feed on blood and transmit microbes that cause disease in their hosts, including humans, domestic animals, and wildlife. Tick cells grown or cultured in the laboratory are tools used in research to better understand tick biology and develop tick control methods. This research adapted three-dimensional (3-D) tissue culture technology for cells derived from the southern cattle fever tick, which transmits the microbes causing bovine babesiosis, or cattle tick fever, that remains a threat to the U.S. livestock industry. The experimental results showed that cells in 3-D culture shifted their shape and aggregated to look more like cells in tick tissues or organs. These findings suggest that 3-D culture could be applied to increase the understanding of tick biology and accelerate research and development of technologies to manage cattle fever ticks.

**Abstract:**

Tick cell culture facilitates research on the biology of ticks and their role as vectors of pathogens that affect humans, domestic animals, and wildlife. Because two-dimensional cell culture doesn’t promote the development of multicellular tissue-like composites, we hypothesized that culturing tick cells in a three-dimensional (3-D) configuration would form spheroids or tissue-like organoids. In this study, the cell line BmVIII-SCC obtained from the cattle fever tick, *Rhipicephalus* (*Boophilus*) *microplus* (Canestrini, 1888), was cultured in different synthetic scaffold systems. Growth of the tick cells on macrogelatinous beads in rotating continuous culture system bioreactors enabled cellular attachment, organization, and development into spheroid-like aggregates, with evidence of tight cellular junctions between adjacent cells and secretion of an extracellular matrix. At least three cell morphologies were identified within the aggregates: fibroblast-like cells, small endothelial-like cells, and larger cells exhibiting multiple cytoplasmic endosomes and granular vesicles. These observations suggest that BmVIII-SCC cells adapted to 3-D culture retain pluripotency. Additional studies involving genomic analyses are needed to determine if BmVIII-SCC cells in 3-D culture mimic tick organs. Applications of 3-D culture to cattle fever tick research are discussed.

## 1. Introduction

Ticks and tick-borne diseases are a serious health burden for humans, livestock, and wildlife [1,2]. The southern cattle fever tick, *Rhipicephalus* (*Boophilus*) *microplus* (Canestrini, 1888), is regarded as the most economically injurious tick affecting livestock because of direct effects on hosts of its obligate blood-feeding habit and its role as a vector of the causative agents of bovine babesiosis and anaplasmosis [3,4]. Control of *R. microplus* infestations in livestock relies on the intense use of chemical acaricides [5]. However, populations of *R. microplus* worldwide evolved resistance mechanisms to virtually all commercially available acaricides [6,7], which highlights the need to develop new tick control technologies [8,9]. The culture of tick cells is an approach that facilitates the research and development of tick control technologies that could also mitigate the burden of tick-borne diseases, including those associated with *R. microplus* [10,11].

Scientific and technological advances enhanced the use of cell lines for research to investigate tick cellular and pathogen interactions, acaricide resistance mechanisms, identification of novel tick-borne agents, and the adaptation of novel antitick vaccine development methods [10,11,12,13,14,15,16,17,18,19]. In the case of *R. microplus*, this involved cell lines derived from embryonic tissues [10,12,20,21,22,23,24,25,26]. Mechanisms contributing to resistance to organophosphate acaricides in *R. microplus* populations were analyzed in vitro by comparing relevant physiological processes between organophosphate-resistant and susceptible cell lines [27,28]. In vitro selection of a BME26 cell line allowed studies that revealed the contribution of ABC transporters to the mechanism of resistance to ivermectin in *R. microplus* [18,29,30]. Research with *R. microplus* cell lines also expanded knowledge of the genetic basis of resistance to amitraz [31]. Additionally, the use of cells lines helped isolate, grow, and study tick-pathogen interactions [10,12,32,33,34,35] and elucidate intricate aspects of biochemical pathways in *R. microplus* [11,36,37,38,39,40]. Technologies for the genetic manipulation of *R. microplus* have been developed using homologous tick cell culture [26,41], and research to apply CRISPR/Cas systems and other technologies to ticks and other Acari is underway [42]. Two-dimensional (2-D) cell cultures were used in these studies, which reflect cellular processes of individual cells that may not represent tissue or organismal responses.

Advances in three-dimensional (3-D) cell culture to produce spheroids and organoids provide advantages in biomedical research that more closely resemble those in tissues and whole organisms [43,44]. 3-D culture systems provide biomimetic microenvironments enabling resident cells to function like cells more accurately in tissues compared to 2-D systems [45,46,47,48,49,50,51]. 3-D cell culture models were developed for biomimicry of native tissue architecture and disease conditions [52,53,54,55,56]. Development of novel biomaterial coated culture plates, sphere culture, bioreactors, and microfluidic systems improved 3-D cell culture platforms [57,58,59,60,61,62,63]. Organoids produced through 3-D cell culture are defined as cells displaying cellular architecture beyond 2-D exhibiting self-organization mimicking the shape and/or function of parental tissues [64,65,66,67,68,69,70]. The benefits of applying 3-D cell culture methods to research with *R. microplus* cell lines remain to be fulfilled [71].

This study describes experiments to bioengineer 3-D in vitro microenvironments with *R. microplus* BmVIII-SCC cells. Different scaffolding systems were used with bioreactors in attempts to grow cells with 3-D architecture. The genesis of tick tissue-like cellular aggregates was achieved with each system tested. Morphologies suggestive of the 3-D structure were identified in cellular aggregates. Prospective research applications of 3-D *R. microplus* cell culture are discussed.

## 2. Materials and Methods

### 2.1. Tick Cell Line

The *R.* (*B.*) *microplus* VIII-SSC (BmVIII-SCC) cell line used in this study was developed at Texas A&M University [22]. This tick cell line was in passage 13 when sourced from the USDA-ARS Knipling-Bushland U.S. Livestock Insects Research Laboratory in Kerrville, TX, and is available from the Tick Cell Biobank at Liverpool, UK [10]. Cells were cultured at 30 °C in a humidified 5% CO_2_ atmosphere incubator.

### 2.2. Cell Culture Medium

A modified Bhat–Yunker’s medium [22,72] was used in all the experiments. The medium consisted of a 1:1 mixture of Leibovitz-15 (Sigma-Aldrich, St. Louis, MO, USA) and Hanks MEM (Sigma-Aldrich) media supplemented with 20% heat-inactivated fetal bovine serum (FBS, Sigma-Aldrich), 10% tryptose phosphate broth (Sigma-Aldrich), 0.1% bovine serum albumin fraction 5 (Sigma-Aldrich), 1% antibiotic/antimycotic (200 U/mL penicillin, 50 mg/mL streptomycin and 50 µg/mL amphotericin B; Sigma-Aldrich), and 200 units/mL ciprofloxacin (Sigma-Aldrich). The medium was adjusted to pH 6.9 with 0.1 M NaOH (Sigma-Aldrich), filter sterilized, and pre-equilibrated by incubation at 30 °C in a humidified 5% CO_2_ atmosphere prior to medium exchange or subculture. The conditioned medium consisted of a complete medium removed from BmVIII-SCC cultures during replacement feeding of the cultures.

### 2.3. 2-D Cell Culture

Frozen BmVIII-SCC cells (passage number 13, P13) were thawed in a 37 °C water bath and infused into 6.0 milliliters (mls) of pre-warmed (37 °C) Bhat–Yunker tick media in sterile Primaria^TM^ 25 cm (Corning Bioscience, Corning, NY, USA) tissue culture flask inside a Baker EdgeGard^TM^ Class II A2 biosafety cabinet (Baker and Baker, Stanford, ME, USA). This base BMVIII-SCC stock was serially passaged throughout the study. Fresh pre-warmed medium (50% of medium volume per flask) was exchanged every 4 to 7 days per monolayer confluency. A 3.0–5.0 mL cell slurry was transferred to new culture flasks after every 3rd media exchange, which constituted a new cell passage number (P14–P58). Confluent cells were dislodged by repeated pulses of cell medium using a sterile flexible transfer pipet, and 2.0 mls of suspended cells were subcultured in Primaria^TM^ 25 cm^2^ cell culture flasks at a 1:3 ratio diluted with fresh medium. A 0.5 mL aliquot of transferred cells was stained with 0.4% Trypan Blue (Sigma-Aldrich, St. Louis, MO, USA) for total cell count, and the mortality rate was determined using a Neubauer hemocytometer (VWR, Radnor, PA, USA) viewed on a binocular light microscope (VWR).

Cells in aliquots showing at least 85% viability were seeded into seven Primaria^TM^ 25 cm^2^ culture flasks at 5.0 × 10^5^ cells/mL to determine cell doubling time. Cells were removed from the surface of individual flasks as described above every 24 h for 6 days and collected by centrifugation at 500× *g* for 5 min at ambient temperature (25 ± 1 °C). After removing the supernatant, the cell pellet was resuspended (1:5) in a fresh medium and stained with 0.4% Trypan blue solution (Sigma-Aldrich). Mean cell counts in each flask were enumerated from 10 separate fields of cells for quantification on a Neubauer hemocytometer (VWR). Data were analyzed using a semi-log plot.

### 2.4. 2-D Cell Morphometrics

Aliquots of 5 × 10^5^ cells/mL were seeded into Primaria^TM^ 25-cm flasks to evaluate the cultures on days 5, 10, 15, 20, 25 and 30 post-inoculation. All 2-D cell cultures were examined on an Olympus CKX-41 Inverted Microscope (Olympus USA, Clear Valley, PA, USA). Cell morphometrics was assessed with a calibrated Olympus Ocular micrometer. Bright-field and polarized photomicrographs were acquired with a Canon EOS -T31dlr (Canon USA, Melville, NY, USA) camera attached to the microscope. Mean morphometric value, standard deviation (SD), and range were calculated from 10 individual measurements of each cell type from 5 separate 40 × fields of view on the Olympus Inverted Microscope on day 30.

### 2.5. 2-D Cell Histological Evaluation

BmVIII-SCC cells propagated in Primaria^TM^-25 cm flasks served as controls to culture 2-D cells on flat plastic surfaces. At confluency, the flasks were rinsed 3 times with phosphate-buffered saline (PBS, Sigma-Aldrich), fixed with 4% phosphate-buffered formalin (Diatome-EMS, Hatfield, PA, USA), and the monolayer disrupted with rubber policemen for transfer to glass slides coated with poly-L-lysine (Sciencell Research Laboratories, Carlsbad, CA). Slides processed at Vel-Labs Research (Missouri City, TX, USA) for Hematoxylin and Eosin (H&E), Masson’s Trichrome, and Periodic Acid—Schiff (PAS) histological staining were evaluated individually at the Cell Systems-3D laboratory for cell morphology and intracytoplasmic structure, lipid, carbohydrate, actin, and cytokeratin as described below.

### 2.6. 3-D Tick Cell-Tissue Culture

*R.* (*B.*) *microplus* BmVIII-SSC cells were infused into these culture microenvironment permutations: (1) nanofiber disks, (2) hydrogels, and (3) gelatinous microbeads using bioreactors consisting of a rotating cell culture system (RCCS). These in vitro culture methodologies were evaluated for: (i) 3-D cell propagation, (ii) cell aggregation, (iii) cellular organization, and (iv) morphometric development into embryonic tick tissue.

#### 2.6.1. Nanofiber Disks

Polyethylene terephthalate (PET, Dacron^TM^), polycaprolactone (PCL), and polyurethane (PU) electrospun synthetic nanofiber disks (Nanofiber Solutions, Columbus, OH, USA) were placed in 12-well Falcon tissue culture plates (Corning Life Sciences, Corning, NY, USA). Disk inserts obtained from the manufacturer had nanofibers aligned in a fixed orientation and fit the diameter of each well. Following hydration, 2 mL of BmVIII-SCC cell-free conditioned medium was added to individual wells containing the disk insert. The plate was incubated overnight at 30 °C in a humidified 5% CO_2_ atmosphere. Two hours (h) before tick cell aliquot infusion, the cell-free conditioned medium was removed and replaced with a 2.0 mL fresh complete medium (medium with FBS, antibiotic/antimycotic solution). The tissue culture plates returned to the incubator for medium equilibration.

BmVIII-SCC cells in Primaria flasks were resuspended as described above, and a loose cell pellet was adjusted to infuse 1.0 × 10^6^ BmVIII-SCC cells in 500 µL complete medium per nanofiber disk. The first complete medium exchange was at 48 h post-infusion (p,i.), allowing the infused cells time to distribute and attach to individual nanofibers. Subsequent medium exchanges were adjusted to a 96 h cycle with 1.0 to 1.5 mL medium exchange for the initial 30 days, then to a 48 h cycle from day 30 through day 90 relative to increasing cell numbers. Individual cells and cell aggregates in randomly selected nanofiber disks were visualized on days 10 through 90 p.i. with the addition of 500 µL of a 0.5% neutral red solution (Sigma-Aldrich). The stain solution was diluted and removed by the normal exchange of completed medium within all cultures at 48 h.

#### 2.6.2. Hydrogel

A HyStem Hydrogel^®^ HP matrix mix (ESI BIO, BioTime, Alameda, CA, USA) was reconstituted following manufacturer recommendations. The thiol-collagen hydrogel mix was adjusted with cell culture medium to deliver a final 2.0 mL volume mixture of hydrogel-medium cells containing 1.0 × 10^5^ BmVIII-SCC cells in each well of a 12-well plate. The plate was incubated for 1 h at 30 °C in 5% CO_2_ atmosphere, and a 1.0 mL pre-equilibrated medium was added to each well. Medium (1 mL) in the hydrogel cultures was exchanged every 48 h for 40 days and incubated at 30 °C in a 5% CO_2_ atmosphere.

#### 2.6.3. RCCS Bioreactors

##### Bead Preparation for Bioreactors

Gelatinous microcarrier (Pharmacia Cultispher-G, Sigma-Aldrich), and porous polystyrene microcarrier (Corning^®^ Enhanced Microcarrier, Corning Life Sciences, Corning, NY, USA) beads provided the physical support scaffolds for cell attachment within the rotating bioreactor vessels to conduct side-by-side experiments. According to the manufacturer’s protocol, ten grams of gelatinous microcarrier beads and 10.0 g of polystyrene microcarrier beads were hydrated. Prior to cell addition, 5.0 mL hydrated bead slurry was washed 3X with 10.0 mL sterile PBS in a 50.0 mL conical tube, allowing the beads to sediment by gravity between each wash. PBS wash was gently removed without disturbing the bead slurry. After the last PBS wash, 10.0 mL cell-free condition media was added to each bead slurry and incubated for 24 h at 30 °C in a 5% CO_2_ atmosphere. Media was replaced with 10.0 mL fresh cell-free conditioned media for a second 24-h incubation. Within each conical tube, 8.0 mL cell-free conditioned medium on the microbeads was replaced with an 8.0 mL cell-free conditioned medium without disturbing the beads and returned to the 30 °C 5% CO_2_ atmosphere for another 24-h incubation.

##### Bioreactor Setup

**Figure 1:**
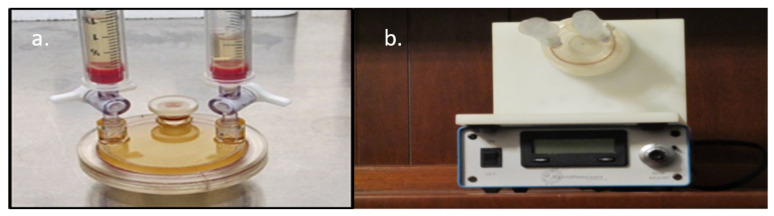
(**a**) Rotating cell culture system bioreactor (10.0 mL) used to develop the 3-D embryonic tick cell models. Syringes are attached to the air purging ports. (**b**) Bioreactor mounted on rotating base and controller.

Cell-free conditioned medium was removed from each conical tube under a laminar flow hood, and 10.0 mL RCCS bioreactors (Synthecon, Houston, TX, USA) were individually infused with a 3.0 mL bead slurry in fresh complete medium (Figure 1), which was followed by the addition of 7.0 mL of cell-free conditioned medium. The bioreactor ports were then closed, air siphoned off with two attached 5.0 mL sterile syringes, and the headspace was filled with medium to avoid air bubbles in each bioreactor. Bioreactors were attached to their rotational base and incubated overnight at 30 °C in a 5% CO_2_ atmosphere at 13 rotations per minute (rpm) for 24 h. Following incubation, 9.0 mL of cell-free conditioned medium was replaced without disturbing the microcarrier beads, and 1.5 × 10^6^ BmVIII-SCC (P32) cells in 1.0 mL fresh complete medium were infused into each bioreactor. Bioreactor vessels were purged of all air, sealed, and reattached to the bioreactor rotational base, which was initially set at 15.0 rpm for 48 h to maximize cell contact and adherence to beads. Individual bioreactor vessel rotational speed was gradually increased to maintain bead-cell aggregates in suspension over the next 90 days of the experiment. As propagation continued, cell-bead aggregations and coalescence of smaller aggregates into bigger ones were observed, which progressed into larger and heavier tick cell-bead aggregates. Maintaining the aggregates in suspension was necessary because suspension lessened the medium vortex shear forces, potentially tearing an aggregate apart. Rotational speed was increased gradually to maintain the larger, heavier aggregates in suspension. Approximately 6.5 mL of complete medium was replaced with a fresh complete medium in both bioreactors every 48 h. The RCCS BmVIII-SCC–microcarrier bead experiment lasted 90 days with a final rotational speed of 27.1 rpm for the Pharmacia Cultispher-G (gelatinous) beads, and 16 rpm for the Corning^®^ Enhanced (polystyrene) Microcarrier beads.

##### Sampling and Microscopic Observation of Cell Aggregates

**Figure 2:**
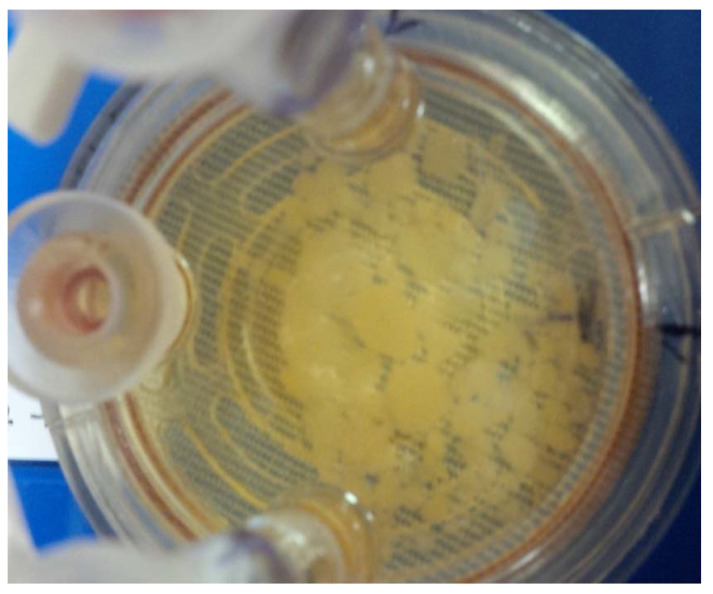
BmVIII-SCC tick cell spheroids grown on macrogelatinous beads in a bioreactor. Day 90 p.i. View: 50X.

Microcarrier bead-cell aliquots (0.5–1.0 mL) from both bioreactors were obtained aseptically every 15 days from day 21 p.i. through day 90 p.i. (Figure 2). Microcarrier-cell aggregates were gently rinsed 3X with PBS, fixed in fresh 5.0–10.0 mL 4% buffered formalin overnight at ambient temperature (25 ± 1 °C), and sedimented to pellets by gravity. One-ml aliquots were embedded in histological tissue cassettes covered with 2% Noble agar (Difco, Becton-Dickinson, Franklin Lakes, NJ, USA) in 2.0 mL warm PBS and allowed to solidify at ambient temperature (25 ± 1 °C). Cassettes were placed in transport containers, covered with 3.0 mL isopropanol, and transported to Vel-Laboratory. All cassettes were embedded in histology-paraffin blocks per Vel-Lab protocol, sectioned at 5 µm with disposable ACCU Edge low profile microtome blades on a Manual Olympus Cut 4055 microtome adhered to glass slides. Sections were stained with Hematoxylin- Eosin (H&E) using Harris Hematoxylin and Eosin Y (Stat-Lab, McKinney, TX, USA), Masson’s Trichrome (Masson Trichrome Stain Kit, Sigma-Aldrich, St. Louis, MO, USA), and Periodic Acid -Schiff (PAS Stain Kit, Master Tech, McKinney, TX, USA). Unstained negative control sections were prepared for comparison at Vel Laboratory. All differential staining was done manually. The immunohistochemical (IHC) staining protocol included a citrate buffer pH 6.0 for antigen retrieval, using slides preheated to 95 °C, which were immersed for 20 min in the citrate buffer and then cooled to ambient temperature.

### 2.7. Immunohistochemistry (IHC)

BmVIII-SSC 4% formalin-fixed cells removed from 2-D Primaria flasks were sectioned and affixed to individual poly-L-lysine coated glass slides. Five µm thick sections of 3-D RCCS cell-microbead aggregates were probed by primary and secondary antibodies labeled with horseradish peroxide (HRP) and developed using diaminobenzidine (DAB) at Vel-Labs. IHC utilized primary antibody prepared against selected cell structural proteins, including 0.05 µg/µL of mouse antipancytokeratin AE1/AE3 (Abnova, Walnut CA), 1:10 dilution of mouse antibovine vimentin (Thermo-Fisher Scientific, Waltham, MA, USA), 1:20 dilution of mouse antichicken cytokeratin AE3 (eBioscience, Affymetrix, San Diego, CA, USA), and 1:200 dilution of mouse antibovine actin (Sigma Aldrich). Antigen activation, antibody labeling, and HRP/DAB followed the manufacturer’s suggested protocols. Evaluation and image capture of individual IHC slides was accomplished using an Olympus CKX-41 Inverted Microscope at the Cell Systems-3D laboratory.

IHC staining of cell-bead aggregates from rotating bioreactors used a manual Shandon Sequenza IHC staining system. All sections were examined by light microscopy with the Olympus CKX-41 Inverted Microscope. Bright-field and polarized photomicrographs were acquired with an attached Canon EOS -T31dlr camera. On day 90 p.i., all remaining cell–microcarrier aggregates were pipetted from each bioreactor, gently washed 3 times in PBS, and fixed for light microscopy immunohistology (IHC) staining or electron microscopy.

### 2.8. 3-D Light Microscopy

Nanofiber and hydrogel 12-well tissue culture plates were observed every 5 days on the Olympus CKX-41 Inverted Microscope to evaluate cell propagation, distribution, and development of defined cell organization-or tissue formation. Light micrographs at X100, X200, and X400 on days 10, 20, 25, 30, and 40 p.i. were obtained at differing focal distances. Images were captured with the attached Canon EOS -T31dlr camera.

### 2.9. Electron Microscopy

Selected PET aligned nanofiber disks and cell-bead aggregates were gently rinsed twice in 5.0 mls PBS for 30 min and fixed in 5.0 mls 3% paraformaldehyde–3% glutaraldehyde in 0.1 M Sodium Cacodylate buffer (Diatome-EMS, Port Washington, PA, USA) overnight at room temperature (25 ± 1 °C). Post-fixation nanofiber samples were transported to Wylie Laboratory, Johnson Space Center, Webster, TX. Samples were gently rinsed twice in 5.0 mls PBS for 30 min and affixed to aluminum stubs with EM specific carbon adhesive (Diatome-EMS) for observation by environmental scanning electron microscopy (E-SEM). E-SEM evaluations used a low vacuum protocol established by the manufacturer (FEI, Hillsboro, OR, USA), and the NASA/Wylie Immunology-Biochemical Analysis Laboratory, Biomedical Research and Environmental Sciences, Wylie—NASA Research Laboratory, Johnson Space Center, Webster, Texas. Data were captured on a FEI Quanta 250 Environmental Scanning Electron Microscope at 10 kV.

Aggregate samples were fixed with 2.5% glutaraldehyde and 2% paraformaldehyde in sodium cacodylate buffer (0.1 M) overnight at 4 °C. Aggregates were transported to the CardioPathology Laboratory, Texas Heart Institute (THI), Houston, Texas, for post-fixation processing according to CardioPathology Laboratory following protocols for transmission electron microscopy (TEM). Aggregates were embedded in Spurs 812 EM-plastic (Diatome-EMS), ultrathin sections cut at 412 nm (silver sections) on a Reichert–Jung Automated Ultramicrotome (Leica Biosystems, Buffalo Grove, IL, USA) with a Diatome Diamond Knife (Diatome-EMS) and fixed on nickel grids (Diatome-EMS). All grids were observed using a Jeol-JSM Transmission Electron Microscope (JEOL-USA, Peabody, MA, USA) at 90kV.

### 2.10. Immunohistochemistry of E-SEM and TEM Prepared Tick Material

Additional analyses were conducted to resolve biophysical and biochemical properties in sheet-like material originally noted by electron microscopy. Aliquots of PET nanofiber-tick cell and tick cell- bead aggregate samples were processed for IHC-SEM, and IHC-TEM evaluation following the procedures described above, except that Protein A-colloidal gold particles were used as the chromophore in efforts to enhance the visualization of cellular-associated material [73,74]. This was done using a Protein A–10 colloidal gold particle (Diatome-EMS) conjugate (1:20) as the secondary chromophore antibody following IHC electron microscopy procedures described above.

## 3. Results

### 3.1. Cell Source for 3-D Culture Experiments

**Figure 3:**
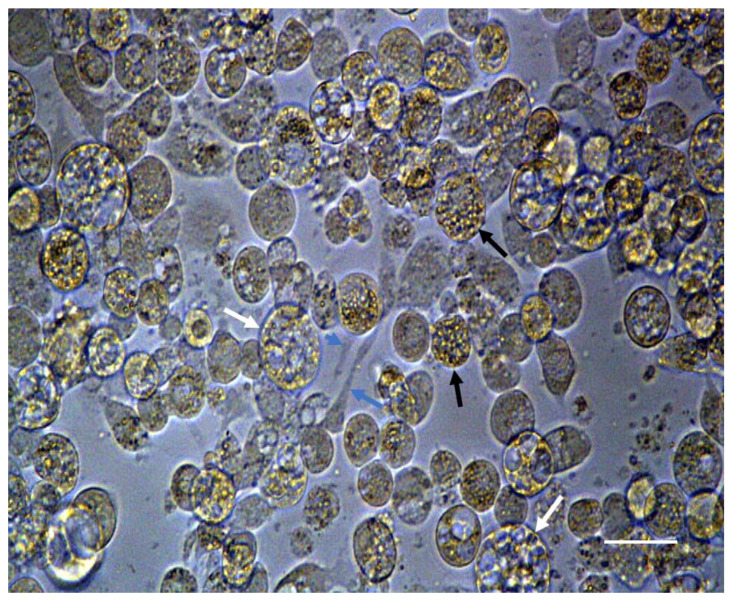
Light microscopy of passage 14 BmVIII-SCC cells cultured in a Primaria flask (View: 400×). Fibroblast-like cells (blue arrows), large vacuolated cells (white arrows), and small granular cells (black arrows). Scale bar: 2.5 µm.

Tick cells utilized for 3-D culture experiments were sourced from the BmVIII-SCC (P13) line propagated in Primaria^TM^ flasks that were subcultured based on monolayer confluency into new flasks between days 20–25 p.i. Cell growth of these subcultures assessed prior to 3-D experimentation provided a baseline for overall cell propagation rate. These data determined the initial cell number for infusion into each cell model and feeding frequency. The overall growth curve for BmVIII-SCC P13 cells cultured in seven individual Primaria T25 flasks indicated a doubling time between 5–6 days (Table 1). On day 30 p.i. under 2-D culture, at least three morphologically dissimilar cell types were observed: fibrocyte-like, small granular endothelial-like, and larger multi-vacuolated cells (Figure 3). Differences in elapsed time to achieve monolayer confluency were not observed in the BmVIII-SCC cell line from P13 through P58 (data not shown).

### 3.2. Hydrogel and 3-D Culture

Measurements of 10 individual cells from 5 separate 40X bright fields were obtained on days 10, 25, 30, and 40 p.i. to assess the effect of 2-D and 3-D culture on the divergent cell morphologies observed. For 3-D culture purposes, the hydrogel was used because it was hypothesized that this matrix would support native conformation and the growth of individual cells. Table 2 shows the size range and mean values calculated for each cell morphology under 2-D culture and 3-D culture with the hydrogel matrix. Measurements for individual fibroblast-like cells could not be obtained in hydrogel cultures on days 10, 25, 30, 40 and small granular endothelial-like and large vacuolate cytoplasm cells on day 10 because unresolved cell orientation within these 3-D culture matrices prevented the quantification of morphological characters.

**Figure 4:**
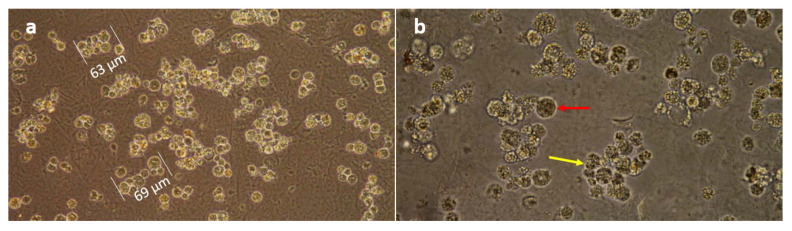
Light microscopy of passage 33 BmVIII-SCC cells cultured with the hydrogel matrix showing aggregate formation. (**a**) Measurements of small granular endothelial-like cell aggregates of “mortar stone” morphology observed on day 25 p.i. at 200×. (**b**) Large vacuolated cells with enhanced intracytoplasmic organization and dense granulation (red arrows), and aggregates of smaller granular cells (yellow arrow) were distributed throughout the matrix on day 30 p.i. as observed at 400×.

Hydrogel culture wells were evaluated by light microscopy for BmVIII-SCC cell proliferation, distribution, and aggregation within the matrix beginning at day 10 p.i. After that, microscopic evaluation occurred every 5 days for the 40-day culture period. Between the small and larger granular-like cells observed, the larger granular-like cells showed cytoplasmic vacuoles and had an endothelial-like appearance (Figure 4). Small granular cells appeared dense (Figure 4a), and aggregated resembling multicellular clusters. Fibroblast-like cells were not observed in the hydrogel cultures. BmVIII-SCC cells were distributed throughout the hydrogel on both horizontal and vertical focal planes. Cells at an angular or transverse orientation could not be clearly determined by light microscopy. Relatively small (12–25 µm) and round granular cells appeared to aggregate when the culture plates were observed dorsally by day 25 post-infusion (Figure 4b). These granular cells displayed a mortar stone-like organization, which morphologically resembled mammalian endothelial-type cells. Although an increase in the number of large vacuolated cells was noted on day 30 pi, by day 40 p.i. a 1:4 ratio of larger to smaller granular cells was observed in the hydrogels.

### 3.3. Nanofiber Disk 3-D Culture

**Figure 5:**
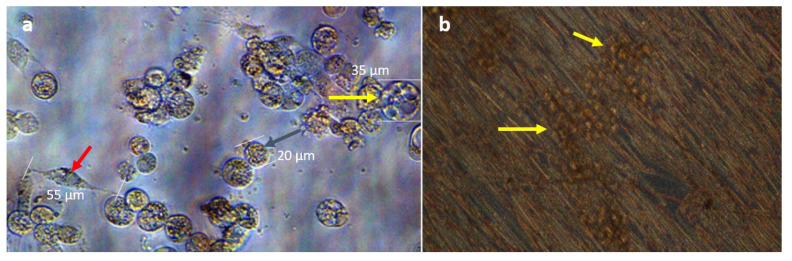
Light microscopy of BmVIII-SCC cells in passage 32 that were cultured in disks with polyethylene terephthalate (PET) aligned nanofibers. (**a**) Luminal orientation day 8 post-infusion of fibroblast-like cells (red arrow), large vacuolated cells (yellow arrow), and small granular cells (dark olive arrow) at 400×. (**b**) Interior view of PET disk on day 35 post-infusion showing cell aggregates (yellow arrows) 200×.

BmVIII-SCC cells adhered to matrices consisting of disks with electrospun PET, PCL, or PU aligned nanofiber mats placed in 12-well culture plates. These aligned nanofibers provided a synthetic support matrix that mimics muscle, bone, and other structural biomatrices in vertebrates. Cells were infused onto the surface of each nanofiber mat and rapidly diffused throughout the ~20.0 µm thickness of each disk (Figure 5a). Cell attachment and propagation occurred throughout the depth of each mat resulting in multiple focal planes (Figure 5b). The number of cells increased during the 90-day observation period, but the clear determination of cytohistology could not be ascertained by optical light microscopy. Because cells are attached in horizontal, vertical, and transverse orientations on individual nanofibers, observations and measurements were limited to cells developing along a horizontal plane. Using a noncytotoxic neutral red (0.5%) stain to deliver a contrast between nanofibers and individual cells was nonproductive because the stain was adsorbed by individual cells, cell aggregates, and synthetic nanofibers. Multiple gentle media exchanges successfully flushed the neutral red stain without observed effects on cell viability.

Cells at the luminal edge of the PET fiber disks were fibroblast-like, small granular endothelial-like, and large multi-vacuolated cells (Figure 5a), comparable to cell morphology observed in 2-D monolayers (Figure 3). In contrast, all three types of nanofibers were initially infused with the same number of BmVIII-SCC cells, observation by light microscopy at 8 days p.i. revealed that the PCL and PU disks retained greater numbers of observable cells at the luminal surface than the PET disks. As time progressed, cell aggregates were observed at different horizontal planes (Figure 5b). Cell aggregates were randomly orientated on multiple focal planes throughout the nanofiber matrix, which hindered precise optical evaluation of a structural organization or cellular enumeration within aggregates by light microscopy.

**Figure 6:**
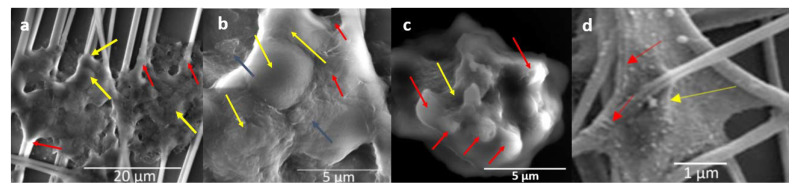
Environmental scanning electron microscopy of cultured BmVIII-SCC cells adhering to PET nanofibers. (**a**) Fibrous-like tissue attaching to individual nanofibers (red arrows) suggests the presence of an extracellular matrix (ECM); note the encased cells (yellow arrows) (3000×). (**b**) Dome-like structures, presumably cells encased in ECM (yellow arrows), flat-sheet morphology of ECM (dark olive arrows), cord-like outer edge of ECM (red arrows) (10,000×). (**c**) Shallow groove (yellow arrow) suggests prior attachment of an enclosed spheroid (red arrows) to a synthetic nanofiber that was dislodged during fixation (10,000×). (**d**) Scanning electron micrograph of the mammalian alveolar cell, A645, in 3-D culture with PET nanofiber provided by Nanofiber Solutions, Inc. (Columbus, OH, USA). is shown for comparison exhibiting fibrous nature of the avian-derived extracellular matrix (red arrows), and structural mass encased by the matrix (yellow arrow).

Images of BmVIII-SCC cells adhering to individual PET nanofibers on day 38 p.i. captured by E-SEM, which minimized cell surface physical distortions associated with alcohol dehydration and critical point drying preparation, showed an attached amorphous, flat sheet-like material, which extended between and attached to multiple nanofiber strands (Figure 6a–c). This sheet-like structure observed in BmVIII-SCC cells resembles the architecture of vertebrate alveolar 3-D cell culture attaching to nanofibers captured previously by E-SEM (Figure 6d), which indicates extracellular matrix (ECM) formation. However, no signal was detected by IHC-SEM using probes with gold-labeled mammalian- and avian-specific pan anticytokeratin, antifibronectin, antilaminin, and antivimentin antibodies. Specific tick histochemical markers were unavailable for this study.

### 3.4. Beads in RCCS

**Figure 7:**
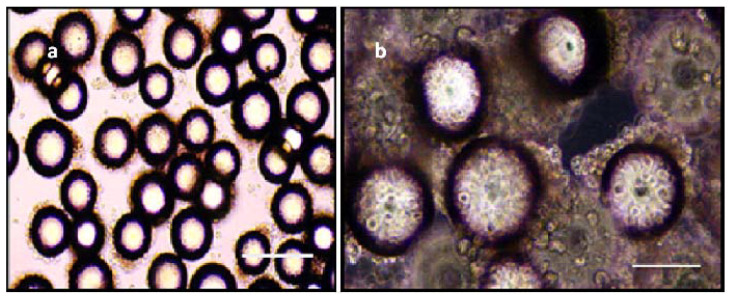
Tick cells grown on polystyrene beads. (**a**) Light micrograph of BmVIII-SCC cells grown with polystyrene microcarrier beads (Corning^®^ Microcarriers) 10 days post-inoculation (p.i.) in rotating cell culture system bioreactor; note early attachment of BMVIII-SCC cells attached along lateral edges of individual beads and the halo-like appearance and hollow core of polystyrene beads that reportedly have a proprietary electrostatic surface charge for improved cell attachment; 100× view; scale bar = 50 µm. (**b**) Light micrograph of BmVIII-SCC cells 30 days p.i. surrounding polystyrene beads; 200× view. Scale bar = 25 µm.

Two RCCS bioreactors, each prepared with the gelatinous and polystyrene microcarrier beads, were seeded with 5.0 mls of tick media for this comparative experiment. In the bioreactor with polystyrene beads (Figure 7a), BmVIII-SCC cells were observed to clump together on day 10, showing friable interactions whereby few cells appeared firmly adhered to beads while maintaining a delicate attachment with adjacent tick cells and bead surfaces. On day 30 p.i., BmVIII-SCC cells covered the outer surface of individual polystyrene beads in structures with a corona-like appearance while exhibiting limited interaction with adjacent cell aggregate-bead clusters (Figure 7b). Bioreactor rotational speeds beyond 16 rpm disrupted cell attachment. Although BmVIII-SCC cells propagated within the bioreactor for the duration of the study, the observed fragility of cell aggregates continued to be a determining characteristic with the polystyrene beads, which presented a challenge when preparing samples for histology and electron microscopy analyses.

**Figure 8:**
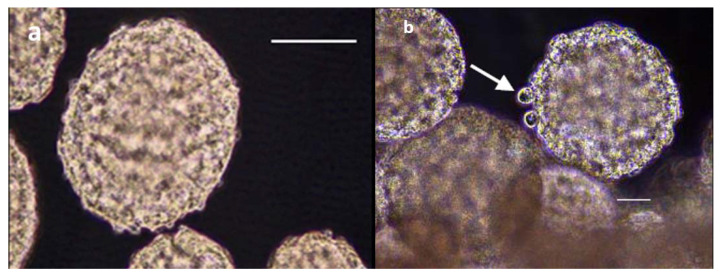
Tick cell growth and aggregation with gelatinous beads. (**a**) Light micrograph of gelatinous microcarrier bead (Cultispher-G^®^) that reportedly has an irregular outer surface with flow-through perforations providing external and internal cell binding locations, which was used to grow BmVIII-SCC cells in rotating cell culture system bioreactor; Scale bar = 12.5 µm. (**b**) Lateral light microscopic view of multiple BmVIII-SCC—gelatinous bead aggregates on day 85 p.i. Membrane-bound and translucent large cell-like structures appeared to bud from bead surfaces covered with cells (arrow). View: 400×. Scale bar = 25 µm.

Compared to the descriptions noted above, contrasting results were obtained when growing BmVIII-SCC cells in the bioreactor using gelatinous beads (Figure 8a). By day 10 p.i., tick cells attached to the surface of individual beads. The number of cells and their organization increased during the study, as evidenced by greater numbers of observable cell-bead aggregates (Figure 2). Cells were observed covering the surfaces of bead aggregates by day 23 p.i. (image not shown). An increase in overall size was observed with time through the attachment of smaller cell-bead clusters that coalesced with the existing aggregates. The surfaces of gelatinous beads that were observed on day 68 p.i. exhibited a mortar stone-like arrangement of small granular cells akin to those observed in mammalian endothelial-like cells (image not shown). Membrane-bound and translucent large cell-like structures appeared to bud from bead surfaces covered with cells (Figure 8b). Depending on aggregate orientation, some cells appeared to be flattened and stacked (MTS personal observation, image not available). In some TEM grids, extrusion or budding of membrane-bound translucent material from the surface of cell-covered beads was noted when viewing from a lateral or transverse aspect.

**Figure 9:**
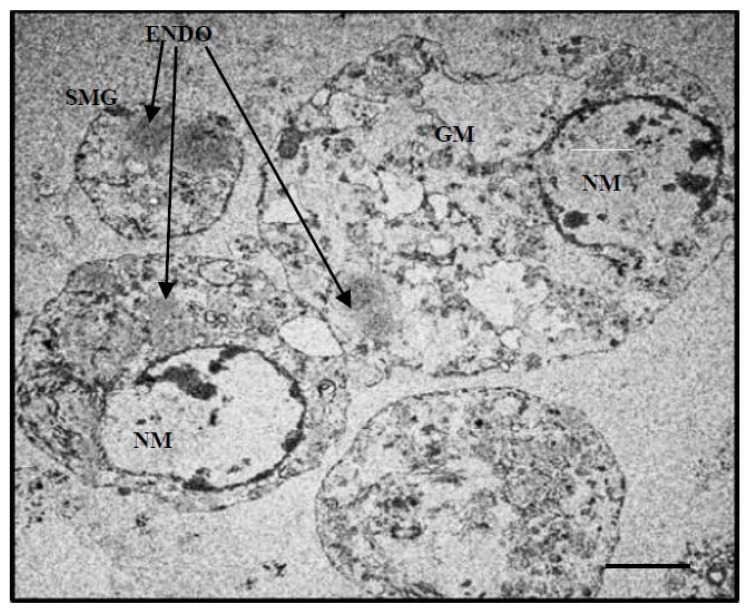
Transmission electron micrograph of a four-cell aggregate of BmVIII-SCC cells from 85-day culture on gelatinous beads in a bioreactor. SMG: small granular cell; ENDO: endosome; NM: nuclear membrane; GM: vesicle granular material. Scale bar = 2.8 µm.

**Figure 10:**
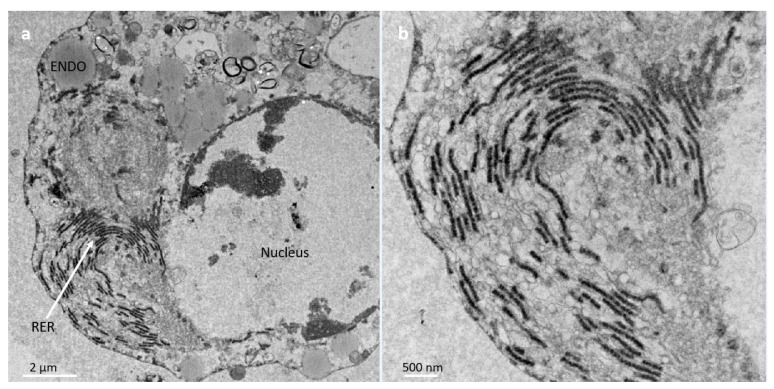
(**a**) Transmission electron micrograph of a large granular BmVIII-SCC cell on day 85 of culture in a bioreactor with gelatinous beads showing perinuclear strands of rough endoplasmic reticulum (RER) within the cytoplasm and membrane-bound vesicles (ENDO), with disrupted nuclear membrane (NM). (**b**) Organized RER showing multiple small vesicles.

Further ultrastructural analysis by TEM of BmVIII-SCC cells in gelatinous gel aggregates on day 85 p.i. revealed small granular and larger multivacuolated cell types surrounded by uniform, granular electron translucent material (Figure 9). As observed by E-SEM (Figure 6), neither defined fibrocyte cell morphology nor fibrous-like covering could be distinguished in the TEM thin sections. However, individual cells appeared to be surrounded by a granular-like material. A cross-sectional view of a four-cell aggregate showed a small granular cell (Figure 9, SMG) and three larger multivacuolated granular cells that exhibited membrane-bound vacuoles with electron-translucent material. The cytoplasm in all cells surrounded thin membrane-bound vacuoles with a translucent granular material (Figure 9, GM). For example, a large granular cell contained relatively large endosomes and multiple membrane-bound vesicles of various sizes together with organized strands of rough endoplasmic reticulum (RER) (Figure 10a). Close inspection revealed multiple small vesicles in close proximity throughout the semicircular rough endoplasmic reticulum, which suggested protein production and Golgi-like packaging into vesicles (Figure 10b).

**Figure 11:**
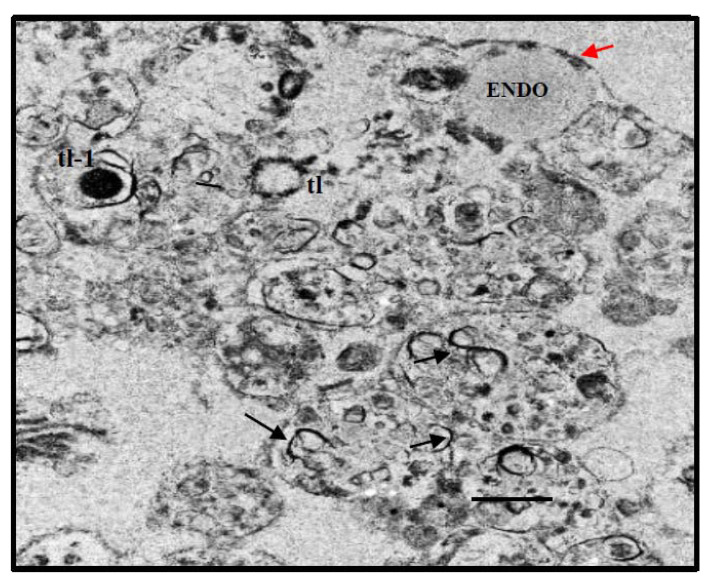
Transmission electron micrograph of a large granular BmVIII-SCC cell on day 85 of culture with gelatinous beads in bioreactor showing membrane-bound cytoplasmic vacuoles with granular material (black arrow), tubular lysosomes (TL), and a large endosome containing electron-dense material (ENDO, red arrow) (Scale bar = 1 µm).

**Figure 12:**
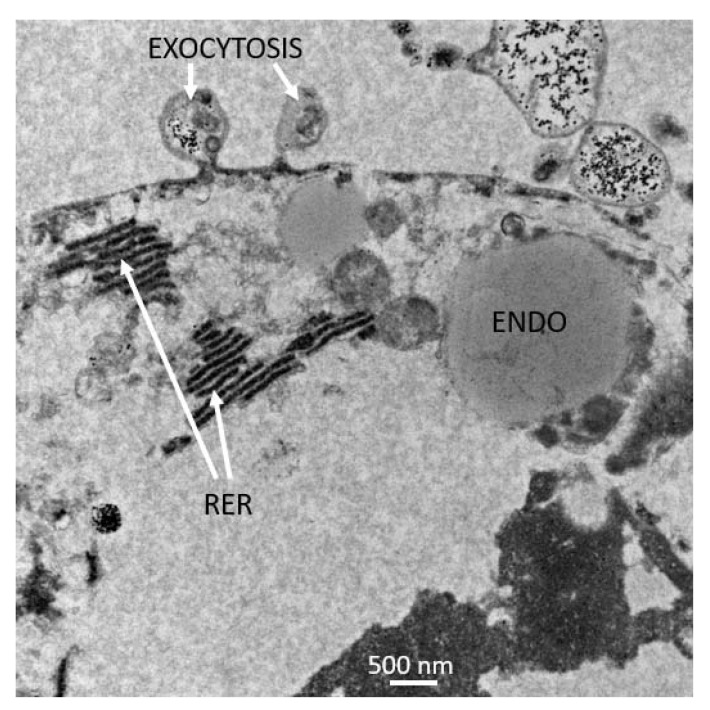
TEM of BmVIII-SCC cell at day 85 p.i. exhibiting budding of cellular material (exocytosis) from the plasma membrane. Rough endoplasmic reticulum (RER) is visible as organized strands. A large endosome (ENDO) is visible, with a smaller endosome located near the site of exocytosis.

Cytoplasmic membrane-bound vesicles with electron-dense granules and electron-dense sigmoid-shaped structures were observed in BmVIII-SCC cells grown in the bioreactor with gelatinous beads (Figure 11). A membrane-bound vesicle filled with electron translucent material was observed in close juxtaposition to the cell membrane. The vesicles with granular material suggest this cell may be a small granular endothelial-like cell. The membrane-bound structure in the upper edge of the micrograph had a uniform distribution of granular material with electron-dense material concentrated and opposed to the membrane (Figure 11). Exocytosis of vesicles containing granular material was noted (Figure 12). Membrane-bound structures containing granular material observed in the photomicrographs could be involved in exocytosis.

**Figure 13:**
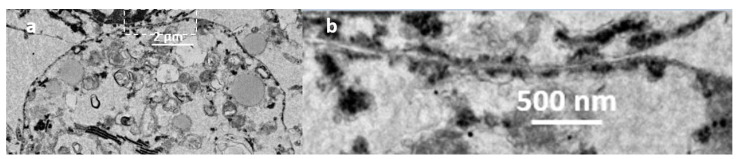
(**a**) Transmission electron micrograph of a large granular BmVIII-SCC cell on day 85 of culture with gelatinous beads in a bioreactor highlighting with demarcation the putative cell-cell junction with another tick cell (**b**) Transmission electron micrograph of a large granular BmVIII-SCC cell on day 85 of culture with gelatinous beads in a bioreactor showing a higher magnification of putative cell-cell junctions with opposed dense regions suspected to include proteins that anchor the cells together and possibly regulate cell-cell transport.

Evidence of cellular structure was observed as electron-dense material at the juxtaposition of neighboring BmVIII-SCC cells grown with gelatinous beads in a bioreactor (Figure 13a). Dense material in what appeared to be a cell-cell lateral gap junction resembled multiprotein complexes known to provide contact or adhesion between cells and control paracellular transport (Figure 13b). However, the cellular organization as defined by an identifiable basement membrane or apical polarity was not observed.

## 4. Discussion

This is the first report of research to develop a 3-D in vitro tick cell culture model. The BmVIII-SCC cell line used in this study was derived from embryonic cells of *Rhipicephalus (Boophilus) microplus* [22]. Many studies have used cell lines under 2-D conditions to research ways to control *R. microplus* [75], which is regarded as the most economically important cattle ectoparasite and vector of bovine diseases globally [76,77]. Characteristics not previously observed under 2-D culture are reported herein after BmVIII-SCC cells were subcultured for development in 3-D microenvironments. Experimental evidence indicated pluripotency, development of cell-cell interactions, and the incipient formation of extracellular matrix-induced in BmVIII-SCC cells grown in a 3-D culture microenvironment. 3-D models of in vivo tissue and organs include spheroids, generally defined as scaffold-free cell clusters that self-assemble or are forced to aggregate from single-cell suspensions. Organoids are self-organized structures derived from cells that mimic the organization and functionality of the tissue from which they are derived [78].

Under 2-D culture, cells of the BmVIII-SCC line were previously characterized as uniformly small and round with a doubling time of 3.8 days [22]. Herein, at least three morphologically distinct cell types are reported during prolonged incubation in 2-D (Figure 3) and 3-D (Figure 4 and Figure 5) culture conditions. These results indicate that the BmVIII-SCC cell line retains a degree of pluripotency. Developmental cytology of the BmVIII-SCC cells appeared to be essentially equivalent when cultured in the 3-D systems tested, except in the synthetic hydrogel microenvironment where no fibrocyte-like cells were observed. Hydrogel matrix formulation has structural, biochemical analogs within the proprietary resin that are activated by a catalyst. Speculating that these biochemical analogs might inhibit the development of a fibrocytic phenotype, the lack of clearly observable fibroblast-like cells in hydrogels may be due to a deleterious effect of the chemical constitution of hydrogels and their finite lifetime to provide sufficient rigidity to support 3-D development. The limited experimental lifespan of hydrogels has been extended by the incorporation of silk chondroitin and other inert materials providing enhanced rigidity and stability [79].

The growth of tick cells in 3-D microenvironments facilitated cell clustering and attachment to one another and to the various synthetic support matrices. TEM micrographs revealed the apparent formation of cell-cell junctions between tightly juxtaposed cells (Figure 13), a morphology known to be induced by a 3-D culture that facilitates cell-cell communication and metabolic regulation [80]. The rough endoplasmic reticulum observed in large granular BmVIII-SCC cells grown with gelatinous beads in a bioreactor (Figure 10 and Figure 12) suggests active protein synthesis, a feature of ECM in 3-D cell culture models [81]. Visualization through E-SEM of small (~3.75 µm × 2.25 µm) dome-like structures delineated by a sheet-like covering (Figure 6) suggested the presence of underlying organized cells. This is the first documentation of evidence for ECM structure in tick cell culture.

Radiating strands of biomaterial attaching to adjacent nanofiber strands (Figure 6) are also suggestive of ECM, which is also observed in association with in vivo mammalian parenchymal tissue [82,83,84,85]. The aforementioned sheet-like material covering underlying cells suggests that cellular interactions and secretion of ECM-like material are facilitated by growth in the 3-D microenvironments, which had not been reported for BmVIII-SCC cells in 2-D culture. Additionally, E-SEM observations were obtained using samples that remained partially hydrated under low vacuum, thereby retaining normal structures, which are unobtainable using high vacuum scanning electron microscopy methods. The purported sheet-like material, covering what may be underlying cells, could provide structural support and points of attachment for the propagating and developing cell mass. However, this sheet-like biomaterial was unreactive when probed with mammalian- and avian-specific pan anticytokeratin, antifibronectin, antilaminin, and antivimentin antibodies. Immunohistochemical probes specific for tick-derived actin, or other fibrous material, are required to determine if this material is of ECM origin. This observation highlights the need for continued research to understand the differences in ECM components between vertebrates and invertebrates [86].

ECM components in *R. microplus* [87], including innexins, remain to be fully characterized. Innexins are proteins that constitute gap junction channels in invertebrates [88]. One putative annexin 2 was identified by mining the transcriptome of *R. microplus* to discover antitick vaccine targets [89]. 3-D tick cell models could facilitate the characterization of ECM proteins and accelerate the functional identification of innexins as targets for controlling ectoparasites, including *R. microplus* [90]. Additionally, further comparative research with 3-D tick cell systems will enhance our understanding of the biomechanical properties of ECM critical to cell-cell communication, migration, and physiological functions, such as stiffness and elasticity of vascular and lymphatic systems [82]. 3-D tick cell culture could also be applied to structural studies of embryonic development where the metabolic pathways for tick vitellin degradation and specific proteolytic enzymes, such as cathepsin B, cathepsin, and acid phosphatase, remain to be fully understood [40,84].

Our experiments with nanofiber disks lasted 90 days, which indicates that these systems provided a suitable matrix for tick tissue development in vitro. Within nanofiber disks, individual synthetic fibers provide the initial structural support for cell attachment and have a product life span of one year. These properties support the relatively slow development of tick cells in vitro compared to hydrogels and other cell systems [91,92]. Nanofiber 3-D mats are available to fit into a variety of multi-well tissue culture plates, potentially allowing the development of simultaneous and high throughput assays that, applied to tick and tick-borne disease research, could be used for acaricidal drug screening, tick-borne pathogen therapies, or antitick vaccine development. Limited cell observation with 2% Neutral Red solution partially overcame a challenge with applying these technologies involving the inability to easily visualize cell aggregate-tissue formation with light microscopy [93]. The potential inclusion of a live/dead stain to visualize cell clusters’ viability and advances in light microscopy technologies, such as optical projection tomography (OPT) and digital holographic microscopy [93,94], may facilitate microscopic observations under these difficult conditions. Super-resolution fluorescence imaging technologies could also be applied to understand the process of tick cell aggregation in 3-D culture systems [95].

Unlike mammalian cells, which have the property of rapid “tissue-like” development, tick cell lines like BmVIII-SCC require longer time spans that generally extend beyond the period of structural stability of existing hydrogel technology, which is approximately 40 days. The introduction of hydrogel formulations with synthetic silk fiber elements or composed of modified or synthetic collagen proteins or other materials [79] may extend their stability, making them more suitable for prolonged 3-D tick cell culture. This will exploit hydrogels’ flexible composition, allowing tick cells to colonize and progress in horizontal, vertical, and tangential orientations, rendering visualization, identification, and morphometric description problematic by standard light microscopy techniques. However, remaining challenges to the wider use of flexible hydrogel matrices for 3-D tick cell culture include incompatibility with standard histological fixation, thick or thin sectioning, and photo-dye labeling using current technology. Advances in microscopy could be applied for the precise evaluation of 3-D tick cell models such as optical projection tomography (OPT) that uses computer-based algorithms to reconstruct composite images from serially acquired images of defined thickness [64,96,97,98,99,100].

As noted for the 2-D culture of BME26 cells [25], further refinements of the media may be required for optimal 3-D culture of BmVIII-SCC cells. Exogenous insulin was reported to stimulate glycogen accumulation in BME26, another *R. microplus* embryonic cell line [101]. Testing permutations of preincubation parameters may also enhance cell adhesion. Dermal human cell 3-D models indicated that alterations in the basal medium formulation in ribonucleotides, amino acids, micro-mineral supplements, Vitamin C, Vitamin D3 influenced the 3-D dermal cells and associated parasites [102,103]. Combining L-glutamine, sodium pyruvate, and insulin improved temporomandibular joint disc tissue cell proliferation without affecting collagen production or gene expression [103]. The sensitivity of fibroblasts to transforming growth factor-β (TGF-β1) was affected by 3-D tissue structure [104,105]. Optimal use of beads for 3-D tick cell culture may require an additional surface attractant, such as chitin or dopamine [106]. Collection of RNASeq data would reveal metabolic changes and identities of gene transcripts expressed, providing indications of cell types and metabolic roles of component cells, i.e., cell identities and tissue-like functions absent in 2-D cultured cells.

3-D cells system applications for drug discovery could be adapted to innovate tick control technologies [107]. Nanofiber 3-D mats are available to fit into a variety of multi-well tissue culture plates, which could be used to advance efforts through panomic approaches [108] to develop high throughput assays for acaricide screening [109], and antitick vaccines [110]. In antigen production, 3-D R. microplus cell systems could be used in bioprinting to accelerate the development of vaccines with high efficacy against different populations of *R. microplus* [111], which is a barrier to the adoption of this control biotechnology around the world.

## 5. Conclusions

This study showed that *R. microplus* BmVIII-SCC cells adhere, propagate and self-organize into 3-D biostructures on synthetic nanofiber disks, hydrogels, and macrogelatinous beads, but not as readily on polystyrene beads in RCCS bioreactors. The relatively rapid growth rate and apparent pluripotency of the BmVIII-SCC cell line offered significant advantages for further 3-D model development. This first-of-a-kind in vitro 3-D tick cell model provides an investigative platform with biomimicry to in vivo tissue for applications in research and development of tick and tick-borne pathogen control technologies. rScaling the multi-well format using nanofiber disks for 3-D tick cell culture could deliver sufficient biomass for panomics studies of tick-borne disease systems.

## Figures and Tables

**Table 1:** Daily means of BmVIII-SCC cell counts over a 6-day culture period after seeding at 5.0 × 10^5^ cells/mL on day 0.

Day	0	1	2	3	4	5	6
Cells/Ml *	5.0 × 10^5^	5.9 × 10^5^	6.4 × 10^5^	7.3 × 10^5^	8.1 × 10^5^	9.1 × 10^5^	1.3 × 10^6^

* Mean cell count was calculated each day by averaging the cells enumerated in ten separate fields of view.

**Table 2:** Measurements for morphologies of BmVIII-SCC cells in 2-D monolayer and 3-D culture with hydrogel matrix.

Cell Morphology	Day 30 (2-D)	Day 10 Hydrogel	Day 25 Hydrogel	Day 30 Hydrogel	Day 40 Hydrogel
Fibrocyte-like	39 µm ± 9.25 µm * (27–55 µm)	N.D.	N.D.	N.D.	N.D.
Small Granular Endothelial-like	16 µm ± 3.25 µm (12–25 µm)	N.D.	18.3 µm ± 1.09 µm (17–20 µm)	18 µm ± 2.07 µm (16–21 µm)	15 µm ± 0.8 µm (14–16 µm)
Large with cytoplasmic vacuoles	33 µm ± 6.65 µm (12–40 µm)	N.D.	31.8 µm ± 5.90 µm (21–34 µm)	24.5 µm ± 2.36 µm (23–33 µm)	36.4 µm ± 2.0 µm (34–39 µm)

* Mean ± standard deviation (measurement range) calculated from measurements (*N* = 15) taken on Day 10, Day 25, Day 30, and Day 40 post-inoculation for cells in 2-D (Primaria flask) and 3-D (hydrogel) cultures. N.D. Not Determined; measurements could not be obtained because of cell orientation within the hydrogel matrix.

## Data Availability

The data presented in this study are available within this publication (Suderman, M.T.; Temeyer, K.B.; Schlechte, K.G.; Pérez de León, A.A. Three-Dimensional Culture of *Rhipicephalus* (*Boophilus*) *microplus* BmVIII-SCC Cells on Multiple Synthetic Scaffold Systems and in Rotating Bioreactors. *Insects* **2021**, *12*, 747, https://doi.org/10.3390/insects12080747).
